# Investigating marine *Bacillus* as an effective growth promoter for chickpea

**DOI:** 10.1186/s43141-023-00608-4

**Published:** 2023-11-24

**Authors:** Khushbu Rathod, Shruti Rana, Pinakin Dhandukia, Janki N. Thakker

**Affiliations:** 1 Department of Biotechnology, P. D. Patel Institute of Applied Sciences, Charotar University of Science and Technology, Anand, Gujarat India; 2 Department of Microbiology, School of Science and Technology, Vanita Vishram Women’s University, Surat, Gujarat India

**Keywords:** PGPB, In vivo studies, *Bacillus licheniformis*

## Abstract

**Background:**

Microorganisms have characteristics that aid plant growth and raise the level of vital metabolites in plants for better growth including primary and secondary metabolites as well as several developmental enzymes. Marine bacteria must endure harsh environmental circumstances for their survival so it produces several secondary metabolites to protect themselves. Such metabolites might likewise be advantageous for a plant’s growth. However, the effectiveness of marine microbes on plant growth remains unexplored. In the present study, we aim to evaluate such marine microbe both in vitro and in vivo as a plant growth promoter.

**Result:**

Marine *Bacillus licheniformis* was found positive for vital plant growth-promoting traits like gibberellin and ammonia production, phosphate and potassium solubilization in vitro. Due to the presence of such traits, it was able to increase germination in chickpea. As it can colonize with the roots, it will be able to help plants absorb more nutrients. Additionally, in vivo study shows that *B. licheniformis* treatment caused rise in vital factors involved in plant growth and development like chlorophyll, POX, phenol, proline, carotenoid, flavonoid, total proteins and SOD which resulted in increase of chickpea height by 26.23% and increase in biomass by 33.85% in pot trials.

**Conclusion:**

Marine *B. licheniformis* was able to promote plant growth and increased chickpea production in both number and weight for both in vitro and in vivo conditions.

**Supplementary Information:**

The online version contains supplementary material available at 10.1186/s43141-023-00608-4.

## Background

The rise in the population across the world requires increase in food production. For this, chemical fertilizers are being used extensively in agriculture, which is essential for production because they supply vital plant nutrients like nitrogen, phosphorous and potassium; these fertilizers have evolved into crucial parts of contemporary agriculture [1]. Yet, excessive fertilizer use may have unintended negative effects on the ecosystem [2]. Thus, it is necessary to reduce the use of chemical fertilizers and boost plant growth in order to improve global agricultural production in a way that is more economically and environmentally sustainable. In more sustainable and environmentally friendly agricultural systems, the use of plant growth-promoting bacteria (PGPB) is a potentially beneficial method for enhancing crop yield [3].

Microorganisms produce plant growth regulators like auxin, cytokinin, and gibberellin, which are utilized by plants and stimulate plant growth [4]. In plants, auxins like indole-acetic acid (IAA) have been noted to trigger both immediate- and long-term responses. They are also called “root-forming hormones of plants” as they increase root length and root hairs and boost development of lateral roots which enables the plant to absorb more nutrients, thus promoting the plant’s total growth [5, 6]. Similarly, gibberellin is reported to promote shoot development including internode extension and apical dominance [7]. These organisms also have the capacity to colonize plant roots, benefiting their hosts by regulating plant hormone synthesis, enhancing the availability of soil nutrients [8]. By the bio-fixation of atmospheric nitrogen and the solubilization of soil minerals, such as phosphorus and potassium, microorganisms operate as a growth promoter, boosting the availability of both macro as well as micronutrients [9].

Generally, soil microbes are studied as plant growth promoters, but marine microbes have received less attention in this aspect. Because of the extreme conditions in the marine environment, which include high pressure, salt, low temperatures, and a lack of light, the marine bacteria have been able to acquire a variety of traits that are not evident in soil or air species [10]. The microbial flora that thrives in the marine ecosystem has adapted to survive in a more demanding environment. Thus, such microbes may produce a wide range of metabolites for its own survival which can be beneficial for plant development in stress condition [11].

*Bacillus* species are highly promising for agricultural usage due to their ability to produce endospores which are tolerant to variety of abiotic stress [12]. Moreover, it is well known that some *Bacillus* species can fix nitrogen, promote the nodulation of other bacteria, and so promote the colonization of rhizobacteria [13]. Here, we aim to study marine *Bacillus* as plant growth promoter as marine microbes have different metabolism than its terrestrial counterpart due to the environmental condition in which it is adapted. Thus, because of this, the metabolites produced by marine microbes may be different to terrestrial counterparts [14] which may be more beneficial for plant growth and development.

For the current study, chickpea is used as it is one of the cheapest protein sources and one of the most consumed foods in the world. It is an essential part of every vegetarian’s diet [10]. As a result, it is a crucial crop for both economic and nutritional reasons. The present study focusses on exploring marine microbe for chickpea growth promotion.

## Methods

### Isolation of marine microorganism and its identification

Marine water sample was collected from Gulf of Khambhat, Gujarat, India (22°30′N,72°61′E). Water sample was collected 10 feet away from seashore, and the depth was 6 feet. Sample was spread on marine agar plates without dilution. For identification, biochemical tests were conducted as per Bergey’s Manual of Systematic Bacteriology, and 16s rDNA sequencing was carried out at Eurofins, Bangalore, India.

### Growth optimization

Optimal growth of organism was checked on marine broth (MB), nutrient broth (NB) and Luria-Bertani broth (LB), varying concentration of salt (0.5 to 8%), pH (5 to 9), temperature (27 to 42 °C) and aeration (100 rpm, 150 rpm and static condition). In all the flasks (having 100 ml media), 100 µl of bacterial culture (10^6^ cells/ml) was inoculated, and every 24 h, 2-ml aliquot was taken from all culture flasks, and its absorbance was measured at 600 nm using Shimadzu UV-Visible spectrophotometer UV-1800. Growth curve was plotted as optical density against time.

### IAA production

Isolate was inoculated in NB with L-tryptophan (200 mg/ml) and incubated for 3 days at 27 °C. Culture was centrifuged, and amount of IAA was measured from supernatant using Salkowski’s reagent. Culture supernatant and Salkowski’s reagent were mixed in equal proportions, and change of colour of supernatant to pink indicated IAA production. Absorbance was measured using Shimadzu UV-1800 UV-Visible spectrophotometer at 590 nm [15]. Standard IAA (HiMedia) ranging from 10 to 100 µg/ml was used to determine concentration of sample.

### Gibberellin production

Isolate was inoculated in NB (100 ml) for 7 days at 30 °C. Culture was centrifuged at 2600 × g for 10 min. Supernatant was used for detection of gibberellin. To 15 ml of supernatant, 2-ml zinc acetate was added and incubated for 2 min at room temperature. After incubation, 2-ml potassium ferrocyanide was added and centrifuged at 2600 × g for 10 min. Supernatant was collected, and 30% HCl was added in equal volume and incubated for 75 min. After incubation, absorbance was measured at 254 nm using Shimadzu UV-1800 UV-Visible spectrophotometer [16]. Gibberellin ranging from 1 to 10 µg/ml was used as standard (HiMedia).

### Phosphate solubilization

Organism was inoculated in Pikovskaya’s agar with 0.05% bromophenol blue and incubated at 27 °C for 5 days. Zone of clearance around colony indicated solubilization of phosphate. Quantitative estimation was done by stannous chloride method. Organism was inoculated in Pikovskaya’s broth (100 ml) and incubated at 30 °C for 5 days. After incubation, 1-ml culture was centrifuged at 2600 × g for 10 min. Supernatant was collected and used for phosphate quantification. In a fresh test tube, 0.1-ml supernatant was diluted with 0.9-ml distilled water. To this, 1-ml chloromolybdic acid (15-g ammonium molybdate was dissolved in 400-ml distilled water, and 400-ml 10-N HCl is added in it with rapid stirring, and total volume was made 1 L with distilled water) and 0.25-ml chlorostannous acid (2.5-g stannous chloride was dissolved in 10-ml concentrated HCl, and total volume was made to 100 ml by distilled water) were added and mixed. Final volume of 5 ml was made up with distilled water. Absorbance was measured at 600 nm using Shimadzu UV-1800 UV-Visible spectrophotometer [10]. Monobasic potassium phosphate was used as standard (HiMedia) ranging from 10 to 100 µg/ml for determination of concentration of sample.

### Potassium solubilization

Organism was inoculated in Alaksandrov’s agar with 0.05% bromothymol blue and incubated at 27 °C for 5 days. Zone of clearance around colony indicated solubilization of potassium. Quantitative analysis was done by inoculating organism in Alaksandrov’s medium (100 ml) having potassium aluminosilicate as the sole potassium source and incubated at 27 °C for 7 days. For quantitative assay, 1-ml sodium cobaltinitrite (1 M) was added to 1-ml supernatant and incubated at 37 °C for 40 min; further, it was centrifuged at 2700 g for 10 min. A total of 10-ml concentrated HCl was added to the pellet resulting in development of blue to green colour. Absorbance was measured at 623 nm using Shimadzu UV-1800 UV-Visible spectrophotometer [17]. Potassium chloride was used as standard ranging from 10 to 100 µg/ml.

### Zinc solubilization

Isolate was inoculated on nutrient agar having 0.1% zinc oxide. Plates were incubated at 27 °C for 3 days [18]. Solubilization index was calculated using the following:

$$Solubilization\ index=\frac{Zone\ of\ solubilization\ (cm)}{Colony\ diameter\ (cm)} \times 100$$

### Ammonia production

Culture was inoculated into peptone water broth (0.2% peptic digest + 0.05% NaCl) and incubated for 5 days at 27 °C. Culture was centrifuged, and to this 200 µl of supernatant, 1 ml of Nessler’s reagent was added. This was then diluted with 8.5 ml of autoclaved distilled water. Brown colour development confirms the presence of ammonia. Absorbance was measured at 450 nm using Shimadzu UV-1800 UV-Visible spectrophotometer [19]. Ammonium sulphate was used as standard ranging from 0.1 to 1 µmol/ml.

### Nitrogen-fixing test

To test the isolate for its nitrogen-fixing ability, it was grown on N-free Jensen media (sucrose 20 g, dipotassium phosphate 1 g, magnesium sulphate 0.5 g, sodium chloride 0.5 g, ferrous sulphate 0.1 g, sodium molybdate 0.005 g, calcium carbonate 2 g, agar 15 g per 1000-ml distilled water) for 2 days at 27 °C. Growth in plates is considered positive for nitrogen fixation [20].

### Siderophore production

MM9 media was defferated as follows. In 100 ml of MM9, minimal media 0.1-M Tris HCl was added, and pH was adjusted to 6.8. In this 100 ml, 0.25% 8-hydroxyquinoline (prepared in chloroform) was added, and this mixture was mixed vigorously in separating funnel. Chloroform layer was discarded, and media was washed twice with chloroform. This defferated media was used to prepare agar plates for qualitative estimation. In this agar plates, 10% chrome azurol S (CAS) was added as an indicator. Organism was inoculated and incubated at 27 °C for 5 days. Orange halos present around colony confirms production of siderophore [21].

### HCN production

Organism was streaked on nutrient agar plates with 5% glycine. Filter paper dipped in 2% sodium carbonate (prepared in 0.5% picric acid) was placed inside of lid and incubated for 5 days at 27 °C after sealing with parafilm. Change in colour of filter paper from yellow to brown indicates HCN production [22].

### Root colonization

Sand was placed inside a sugar tube up to a depth of 6 cm, and then soil was placed on top, up to a depth of 4 cm, and autoclaved. The bioformulation of BS 94 (10^6^ cells/ml) was applied after surface sterilization of seeds. These seeds were placed in an autoclaved sugar tube with sand and soil that was then parafilm sealed. After 10 days, saplings were uprooted, their roots were removed carefully with the use of sterile forceps, and they were then inoculated on nutrient agar plates to check for bacterial colonization [23].

### Water agar test

Organism was spread on water agar plates (1% w/v) and incubated for 7 days. Five healthy surface-sterilized chickpea seeds were randomly chosen and placed on a water agar plate, with BS 94 and without BS 94 (Control). It was incubated at 30 °C for 5 days [24]. Germination percentage and vigour index were calculated by following formula:

$$Germination\ percentage=\frac{Number\ of\ seeds\ germinated}{Total\ number\ of\ seeds} \times 100$$

$$Vigour\ index=Germination\ percentage \times 100$$

### Pot trials

Chickpea seeds of the hybrid desi type procured from local market were sterilized using distilled water for 1 min, 70% methanol for 1 min, followed by 30-s rinse in distilled water. Bioformulation of BS 94 was prepared using talc, calcium carbonate and carboxymethyl cellulose (CMC). In a sterile metal tray, 15 g of calcium carbonate per kg of talc and 10-g CMC per kg of talc were mixed and autoclaved [22]. This autoclaved talc and 10^6^ cells/ml of BS 94 were used to prepare coating slurry. Seeds were coated with it, and ten seeds were sown per pot. Silty clayey loam soil having pH 8 was filled in each pot. Treatments given were as follows: T1—control (untreated seeds) and T2—BS 94 (seeds coated with BS 94 bioformulation).

The pot experiment was conducted in June, when temperatures ranged from 29 to 32 °C using randomized block design with three replicates. After 28-day vegetative parameters, SOD, POX, proline, phenolic compounds, chlorophyll, carotenoid content [25], flavonoid [26], and total proteins [27] were estimated.

### Flavonoid estimation

Total flavonoids were estimated by aluminium chloride method using quercetin as standard. Leaf sample (5 g) was crushed in 10 ml of methanol and centrifuged at 2000 g for 10 min. Supernatant was transferred in new tube and stored at 4 °C. For flavonoid estimation, 125 µl of extract was added to 75-µl 5% sodium nitrite. The mixture was incubated for 6 min at RT. After incubation, 150-µl aluminium chloride was added and incubated for 5 min at RT. After incubation, 750-µl 1-M sodium hydroxide was added, and final volume was adjusted to 2.5 ml with distilled water, and absorbance was measured at 510 nm using Shimadzu UV 1800 UV-Vis spectrophotometer [26].

### Total protein

Total protein was determined using Folin-Lowry method using bovine serum albumin (BSA) as a standard. Sample (5 g) was crushed in 5-ml sodium phosphate buffer and centrifuged at 2000 g for 10 min. Supernatant was stored at 4 °C. To 1 ml of sample, 4 ml of aluminium copper sulphate (Reagent C) was added and incubated for 10 min at RT. After incubation, 0.5 ml of Folin–Ciocâlteu reagent was added and incubated for 30 min in dark. After incubation, absorbance was measured at 660 nm using Shimadzu UV 1800 UV-Vis spectrophotometer [27].

### Statistical analysis

All the in vitro quantification assays were done in triplicates. Post pot trials analysis of variance (ANOVA) was performed using triplicate data to find the significant differences between treated and untreated seeds in each vegetative parameter and enzyme assay. Results are presented as mean ± SD.

## Results

**Fig. 1:**
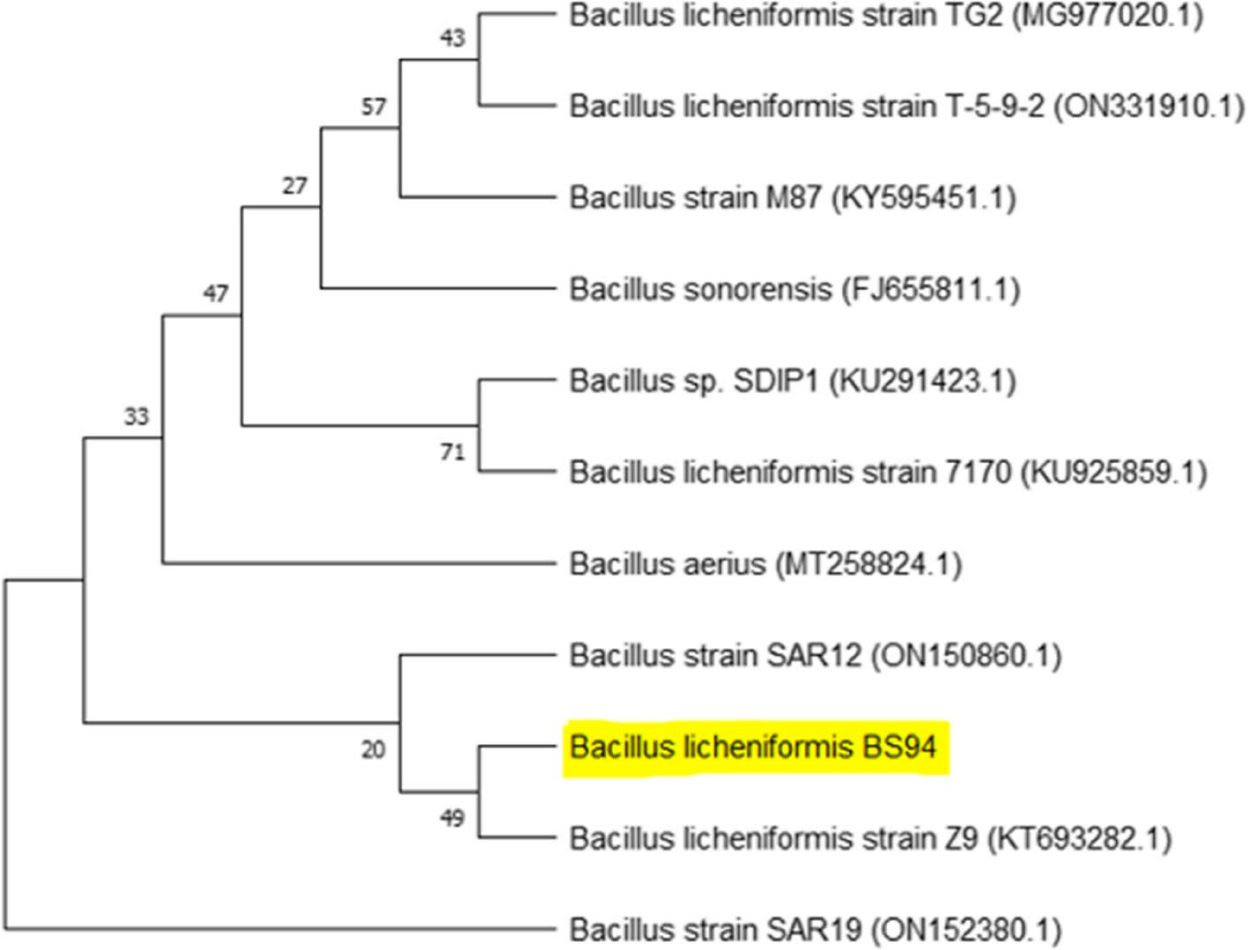
Phylogenetic tree of BS 94 showing maximum similarity with *Bacillus licheniformis* strain Z9

Bacterial strain 94 (BS 94) was isolated from marine water. BS 94 was able to grow on marine agar, nutrient agar, and Luria-Bertani agar, forming white colonies. Table 1 displays the outcomes of biochemical tests. 16s rDNA sequencing was conducted at Eurofins, Bangalore (Fig. 1). The BS 94 sequence was submitted to GenBank, and accession number assigned is OQ519861. BS 94 displayed the highest degree of similarity with *Bacillus licheniformis* strain Z9 under accession number KT 693282.1.

**Table 1:** Biochemical test of BS 94

Test	BS 94
Gram staining	+
Endospore staining	+
Motility	+
Salt tolerance	12%
Growth at 50 °C	+
Oxidase	-
Catalase	+
Gelatinase	-
Citrate utilization test	+
VP test	+
Nitrate reductase test	+
Urease test	+
MR test	-
Casein hydrolysis	+
Starch hydrolysis	-
Triple sugar test	+
Lipase	-
Hemolytic activity	-
Cellulase activity	+

**Fig. 2:**
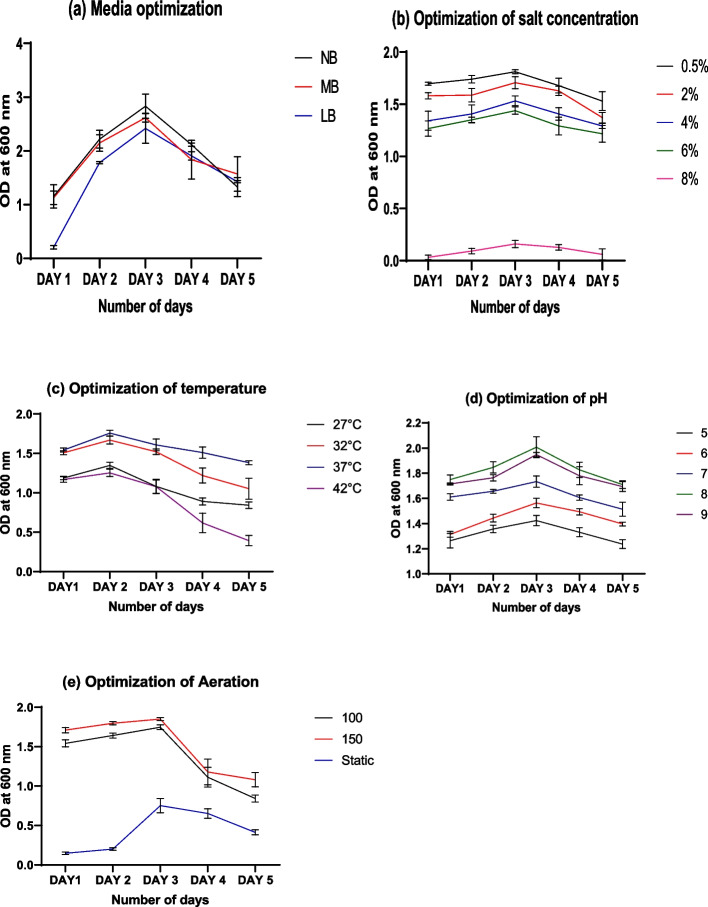
Optimization of growth of BS 94 at different **a** media, **b** salt concentration, **c** temperature, **d** pH, and **e** aeration

As shown in Fig. 2, BS 94 was grown under various conditions to optimize its growth. The average value of triplicates is plotted in the graph where the error bar shows standard deviation. In comparison to other media, nutrient broth promoted the highest growth of BS 94 (Fig. 2a). In response to this, nutrient broth was used for all testing. Best growth was demonstrated at 0.5% concentration of salt (Fig. 2b), 8 pH (Fig. 2c), 37 °C (Fig. 2d), and 150-rpm aeration (Fig. 2e).

**Fig. 3:**
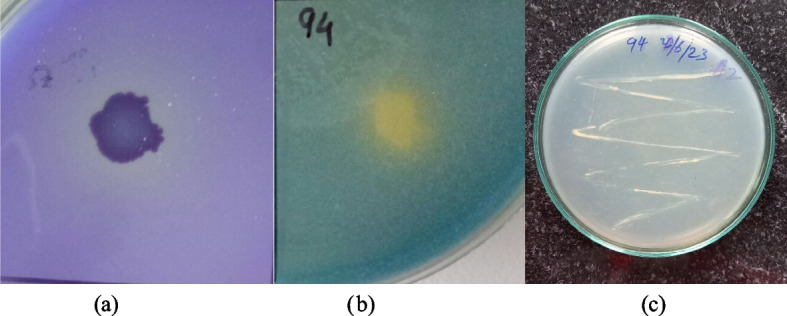
**a** Phosphate solubilization. **b** Potassium solubilization. **c** Growth in Jensen media indicating Nitrogen fixing ability

Isolate did not produce IAA, but gibberellin production of 14.2 µg/ml was noted after 7 days. Solubilization indices of 1.31 ± 0.03 for phosphate (Fig. 3a) and 1.41 ± 0.02 for potassium (Fig. 3b) were found positive respectively. Its quantitative analysis showed 16.23 µg/ml of potassium and 26.86 µg/ml of phosphate solubilization by the isolate. Peptone water was used to test the ammonia production. After 3 days, BS 94 produced 0.45 µmol/ml of ammonia. BS 94 was able to grow in nitrogen-free Jensen media (Fig. 3c) which shows its ability to transform atmospheric nitrogen into fixed nitrogen, an inorganic form of nitrogen which can be easily utilized by plants. BS 94 showed no HCN and siderophore production nor zinc solubilization.

**Fig. 4:**
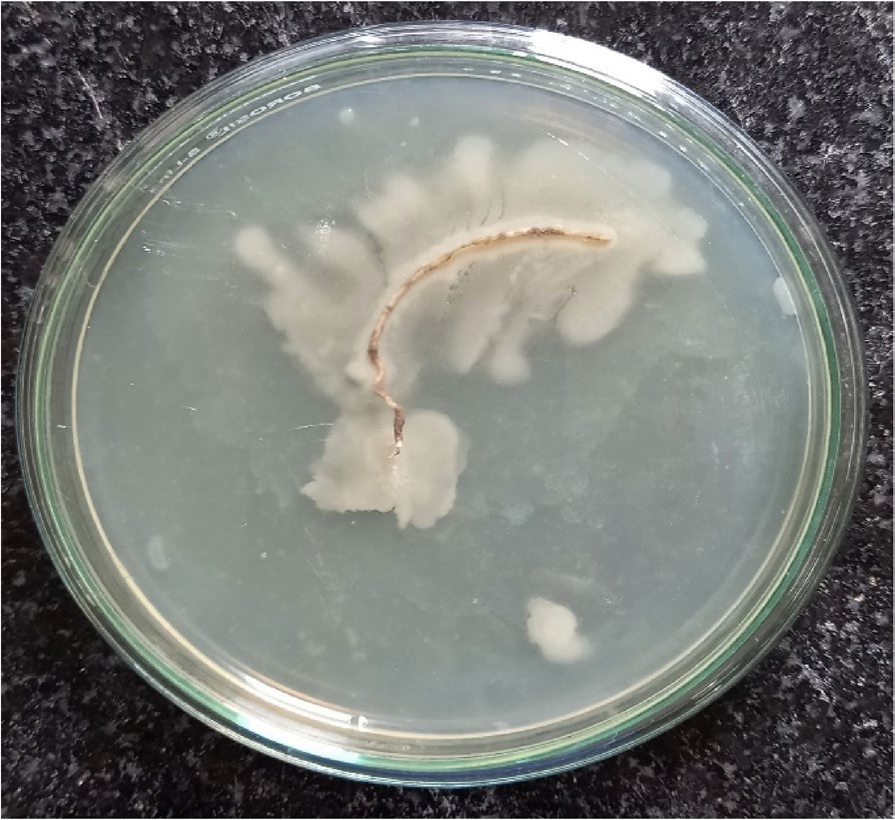
Root colonization of *Bacillus licheniformis*

**Fig. 5:**
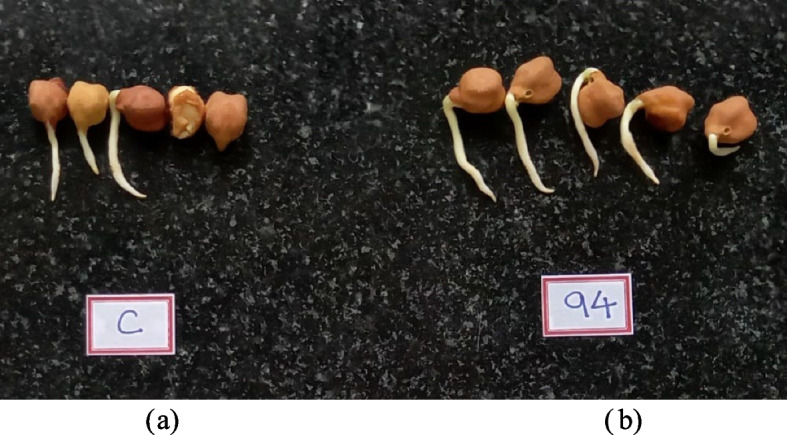
Germination in chickpea seeds. **a** Control. **b** Isolate 94

BS 94 was capable of colonizing roots which was confirmed after 7 days of incubation (Fig. 4). This test confirms it ability to colonize with root even though it is a marine isolate which may help for germination and other growth parameters. It was also capable in increasing germination in seeds up to 100% (Table 2) along with increasing root length (Fig. 5b) as compared to control (Fig. 5a).

**Table 2:** Percentage germination and vigour index of chickpea seeds

Treatment	% Germination	Vigour index
Control	60%	63.6
BS 94	100%	162

**Fig. 6:**
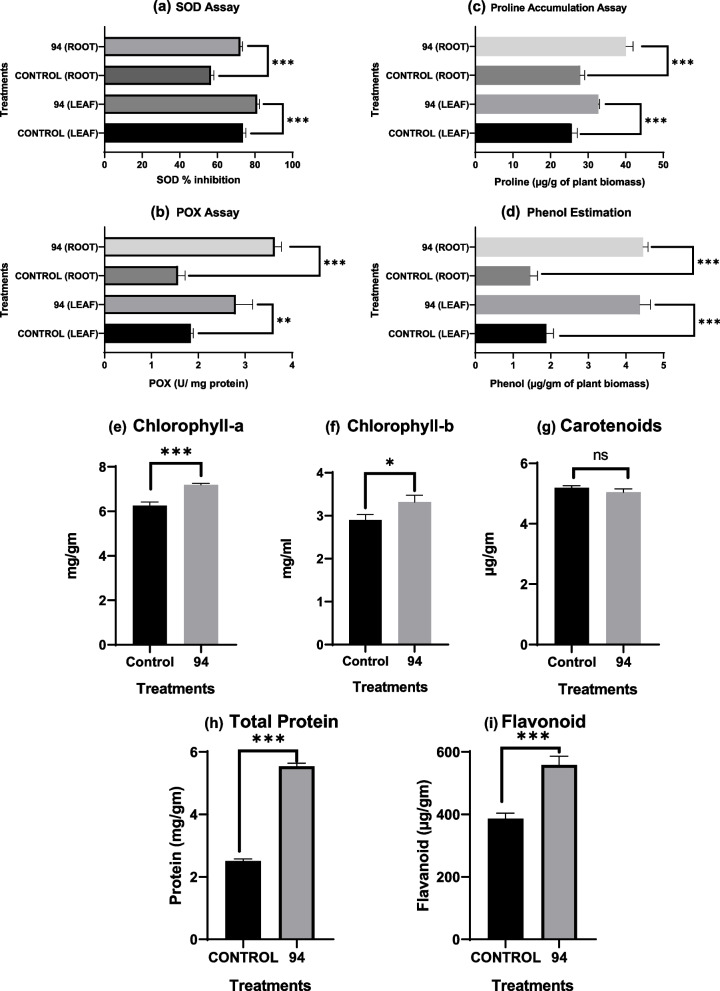
**a** SOD. **b** POX. **c** Proline. **d** Phenol. **e** Chlorophyll-a. **f** Chlorophyll-b and **g** carotenoid. **h** Total protein. **i** Flavonoid content of control and BS 94 post pot trial. Note: *p*-value has been calculated using one-way ANOVA, and its interpretation is as follows: ns (*p*-value greater than 0.05), nonsignificant as compared to control. *p-value between 0.05 and 0.01, significant at 5% as compared to control. **p-value between 0.01 and 0.001, significant at 1% as compared to control. ***p-value less than 0.001, significant at 0.1% as compared to control

BS 94 was examined for in vivo growth promotion in chickpea since it demonstrated traits as a plant growth promoter in vitro. The talc-based BS 94 bioformulation considerably enhanced the vegetative parameters (Table 3) as well as the SOD (Fig. 6a), POX (Fig. 6b), proline (Fig. 6c), phenols (Fig. 6d), chlorophyll-a (Fig. 6e), chlorophyll-b (Fig. 6f), and carotenoid content (Fig. 6g). With the BS 94 treatment, plant mass and height both rose by 33.85% and 26.23%, respectively.

**Table 3:** Vegetative parameters post pot trial

	Control	BS 94
Total length (cm)	39.6 ± 0.754	50.02 ± 1.08^***^
Root length (cm)	3.40 ± 0.16	4.01 ± 0.08^**^
Shoot length (cm)	36.19 ± 0.66	46.00 ± 1.05^***^
No. of leaves	101.33 ± 2.51	108.33 ± 2.08^*^
No. of root hairs	32.333 ± 2.5	37.33 ± 1.15^*^
Total fresh mass (g)	1.27 ± 0.03	1.7 ± 0.21^*^
Shoot fresh mass (g)	1.124 ± 0.036	1.39 ± 0.19^*^
Root fresh mass (g)	0.145 ± 0.005	0.30 ± 0.04^**^
Total dry mass (g)	0.60 ± 0.065	0.69 ± 0.03^*^
Shoot dry mass (g)	0.523 ± 0.066	0.60 ± 0.033ns
Root dry mass (g)	0.08 ± 0.002	0.086 ± 0.003^*^

*p*-value has been calculated using one-way ANOVA, and its interpretation is as follows: ns (*p*-value greater than 0.05), nonsignificant as compared to control

^*^p-value between 0.05 and 0.01, significant at 5%

^**^p-value between 0.01 and 0.001, significant at 1% as compared to control

^***^p-value less than 0.001, significant at 0.1% as compared to control

## Discussion

Bacteria stimulating plant growth, comprises of free living bacteria which can have symbiotic association with plants, and endophytes that can colonize plant's internal tissues. Plant growth-promoting bacteria (PGPB) may either directly stimulate plant development by typically improving mineral utilization or modifying levels of plant hormones or indirectly by reducing the growth-inhibiting impacts of several biotic and abiotic factors [28]. PGPB improves vegetative growth and boosts plant enzyme activity and can support other microbes in a synergistic manner to enhance their impact on plants, encouraging plant growth [29]. Majority of such PGPB studied are from plant’s rhizospheric region, but not much work is reported on marine microbes as plant growth promoters. Present study focusses on exploring marine microbes as plant growth promoters.

Marine isolate BS 94 was identified as *Bacillus licheniformis*. The organism is non-pathogenic as it was tested negative for haemolytic activity (Table 1). Isolate BS 94 being an endospore-forming bacteria will be able to survive in field conditions for longer time. *B. licheniformis* BS 94 was able to grow in wide salt and pH range and temperatures as high as 42 °C as seen in growth optimization (Fig. 2). Marine *Bacillus* isolated from saline soil gave up to 10% salt tolerance [30], whereas *Bacillus licheniformis* BS 94 is able to tolerate up to 12% salt. This organism can therefore be utilized to grow crops in wide pH and salt range and at wide range of temperature. It was also found positive for nitrate reduction, urease and ammonia production. Hence, it could participate in nitrogen fixation. The most essential component for plant development is nitrogen. Ammonia, nitrate and nitrite are three forms of nitrogen that plants can absorb [31]. In most soils, these nitrogen forms are not in great abundance. In these conditions, bacteria’s ability to fix nitrogen plays a critical role. BS 94 was found positive for nitrogen fixation and also produces ammonia which can supply nitrogen to plants and thereby promote height and their biomass. Coinoculation of nitrogen-fixing bacteria *Mesorhizobium ciceri* and mycorrhizal fungi *Glomus* raised chickpea height 23.4% [32], whereas BS 94 treatment alone increases chickpea height by 26.23% (Table 3). BS94 treatment showed increase in total protein content of leaves which may be due to increase in nitrogen as nitrogen present in leaves plays a vital role in protein synthesis [33]. BS 94 was able to colonize roots; hence, it might contribute in growth promotion and stress tolerance in plants [8]. In addition to producing ammonia, BS 94 had the ability to reduce nitrates and produce urease which are the key enzymes in nitrogen assimilation. Urease helps in stimulating hydrolysis of urea into ammonium, which is utilized by plant root [28]. Urease is also involved in bioremediation of heavy metals [34]. Thus, it will be able to reduce plant stress as well. BS 94 produced amylase as it was able to hydrolyze starch. Amylases convert complicated polysaccharides like starch into glucose or sugar that is rapidly utilized by plants and boosts their development [35]. Another vital nutrient for plants is phosphorus. Phosphorus is a component of adenosine triphosphate (ATP), which is produced during photosynthesis and is involved in all stages of plant growth, but it is especially crucial for stimulation of root growth, which enhanced seed development [36]. Although phosphorus is present in bonded form with inorganic or organic molecules, but only the monobasic and dibasic forms of phosphorus can be absorbed by plants. The organic phosphorus is mineralized by BS 94 so that plants can access it. Potassium, the third most important nutrient, is likewise available in insoluble form, much like phosphate. Both phosphate and potassium were shown to be solubilized by BS 94 (Fig. 3). It solubilizes crucial nutrients, which may promote plant growth as indicated by the results of vegetative parameters in Table 3. Root hairs and root length were observed to increase with BS 94 treatment, aiding in increased nutrient absorption and plant support. BS94 was observed to increase secondary metabolites like flavonoids which are reported to assist in uptake of nutrients as well as lignin synthesis in plants which may help in defence [37, 38]. BS 94 was also noted to produce gibberellin which may aid in embryo’s development potential and improves fruit growth and seed germination and induces elongation of root and stem. Seed germination was more in BS 94-treated seeds as compared to control. Marine *Pseudomonas* OG is reported to increase seed germination up to 93.3% in chickpea [39], whereas with BS 94 treatment germination rose up to 100%. Additionally, physiological trait like seed vigour was also increased in treated plants. Seed vigour is essential to promote quick and consistent germination of crops, quality of seed and its performance in field trials [40, 41]. These combined effect of all the nutrients resulted in increase of chickpea height and weight by 26.23% and 33.85% respectively in pot trials after 28 days, whereas marine *Micrococcus* species was able to increase height and weight of chickpea by 19.65% and 21.60% after 30 days [10], and rhizospheric bacteria *Azotobacter chroococcum* treatment resulted in 23.48% increase in height after 30 days [42]. Thus, marine isolate *B. licheniformis* BS94 showed better results than other marine and soil microbe reported earlier.

Along with essential plant nutrients, BS 94 also stimulated enzymes and other factors involved in plant growth and development. Post pot trials, BS 94 showed increase in free radical scavengers like superoxide dismutase (Fig. 6a) and carotenoids (Fig. 6g). These antioxidants guard against cellular membrane deterioration and function as vital signalling molecules that control plant development and stress responses [25]. BS 94 increases peroxidase enzyme in plant which contributes to the production of lignin which increases the rigidity and hydrophobicity of plant cell wall as well as the transport of minerals through vascular bundles in plants [43]. An increase in phenol and proline content was also observed with BS 94 bioformulation which may assist to strengthen plant as phenolic compounds are involved in lignin synthesis and prolines in cell wall formation and abiotic stress. A rise in chlorophyll content in seeds treated with BS 94 was seen post pot study. This may be due to organism’s ability to assimilate nitrogen and potassium solubilization as nitrogen and potassium are major components essential for chlorophyll production along with magnesium and iron. Thus, it may be helpful in improving chickpea production in the field as well.

This study unequivocally proves that the marine *B. licheniformis* had a favourable effect on chickpea’s growth and development. The majority of plant growth promotion research is on rhizospheric bacteria; however, knowledge of PGP characteristics in marine microorganisms can aid in the creation of fertilizers in hitherto unexplored areas.

## Conclusion

In present study, marine organism isolated was identified as *Bacillus licheniformis*. *B. licheniformis* was able to increase height, biomass and several other enzymes and factors which induce growth in plants. Also, as it was able colonize roots and tolerate wide environmental conditions; it can be also used for other crops requiring such growth conditions. But in addition to this study, further investigation into its effect in field conditions and mode of action is needed to develop a potent biofertilizer.

### Supplementary Information

**Additional file 1: Annexure I: Supplementary material Figure S1.** BLAST- N of PCR amplified 16Sr RNA gene sequence of BS94 with published sequences of NCBI database.

## Data Availability

The datasets used and/or analysed during the current study are available from the corresponding author on reasonable request.

## Abbreviations

SOD: Superoxide dismutase
POX: Peroxidase
PGPB: Plant growth-promoting bacteria
IAA: Indole acetic acid
MB: Marine broth
NB: Nutrient broth
LB: Luria-Bertani broth
HCl: Hydrochloride acid
CAS: Chrome azurol S
VP test: Voges–Proskauer test
MR test: Methyl red test
ANOVA: Analysis of variance
ATP: Adenosine triphosphate
