# Supplementary material for: Investigating marine Bacillus as an effective growth promoter for chickpea
Source: J Genet Eng Biotechnol. 2023 Nov 24;21:137. doi: 10.1186/s43141-023-00608-4 (PMC10673802; doi:10.1186/s43141-023-00608-4)
Supplement: Supplementary file 1 — Additional file 1: Annexure I: Supplementary material Figure S1. BLAST- N of PCR amplified 16Sr RNA gene sequence of BS94 with published sequences of NCBI database. [file 43141_2023_608_MOESM1_ESM.docx]

**Annexure –I Supplementary material**


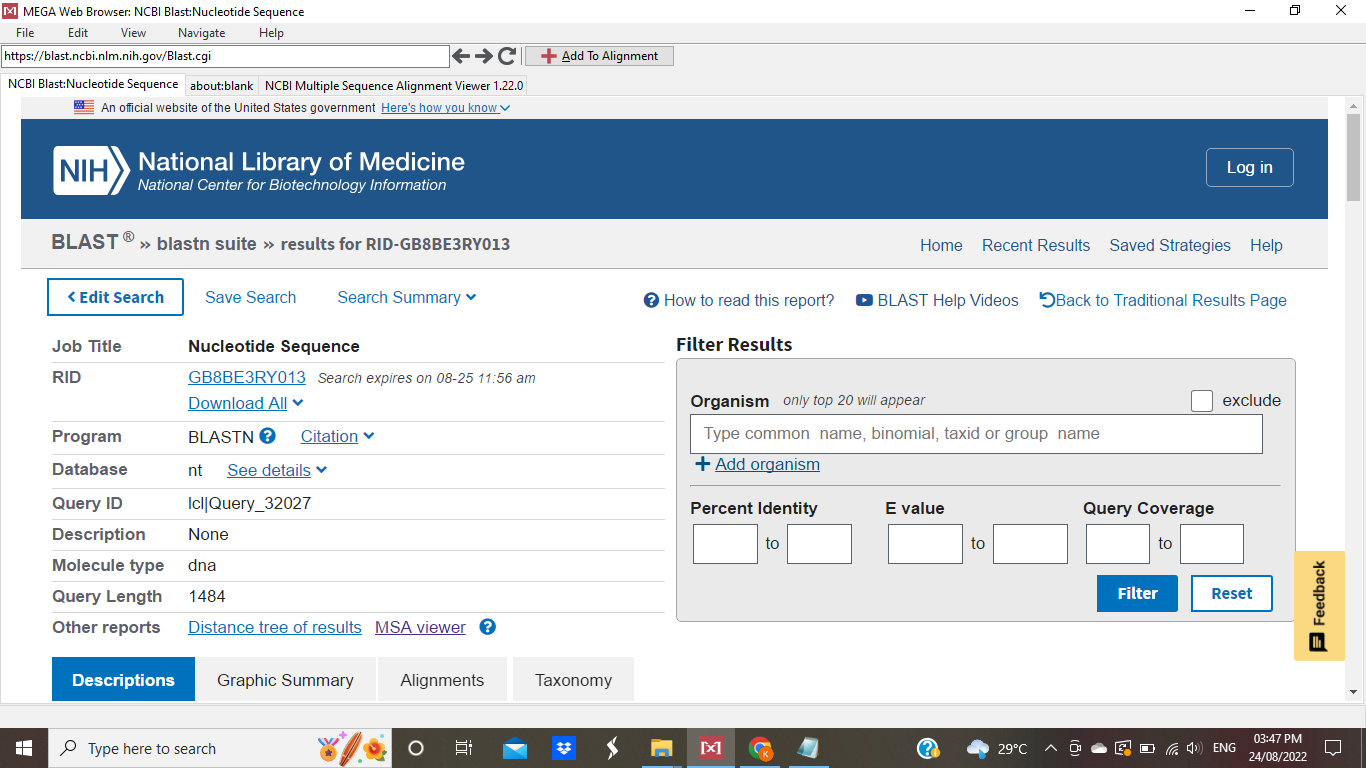


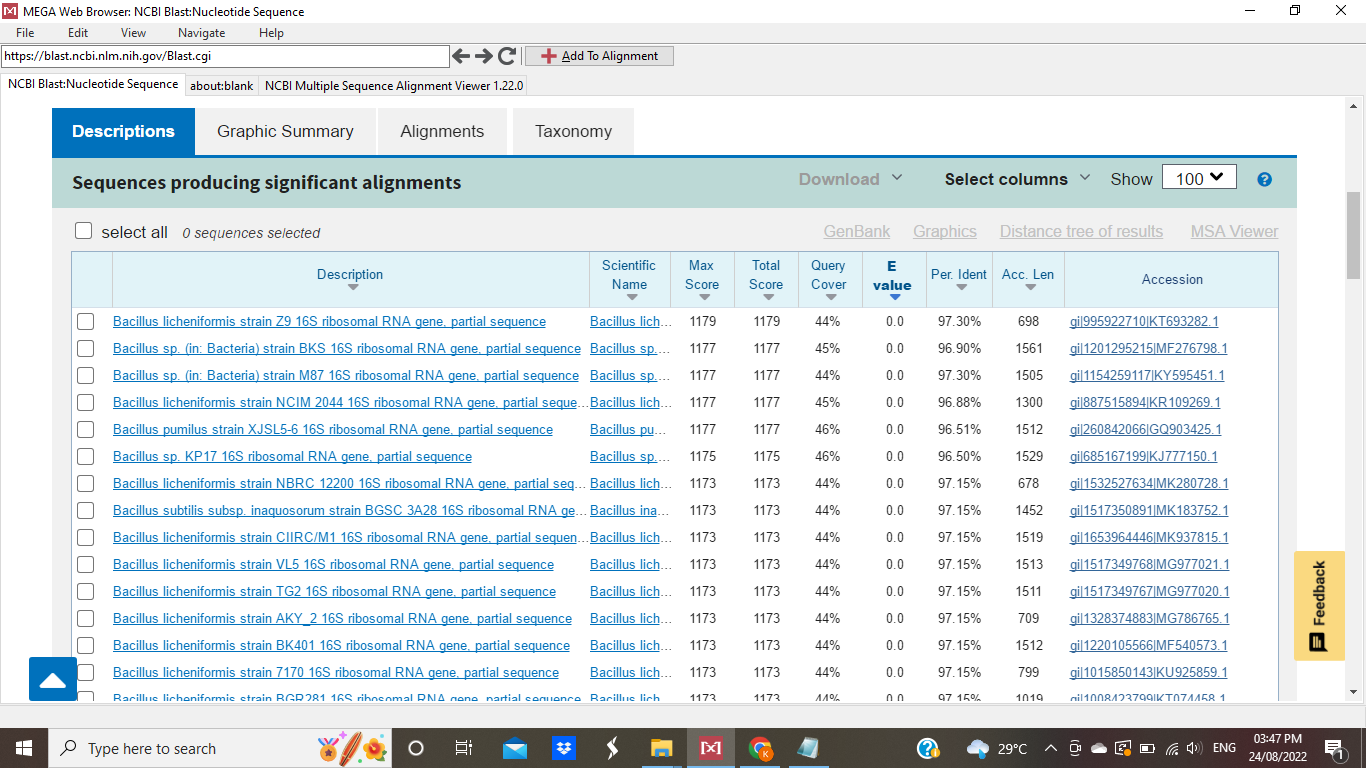


Figure S1: BLAST- N of PCR amplified 16Sr RNA gene sequence of **BS94** with published sequences of NCBI database.
